# Deriving Multiple Benefits from Carbon Market-Based Savanna Fire Management: An Australian Example

**DOI:** 10.1371/journal.pone.0143426

**Published:** 2015-12-02

**Authors:** Jeremy Russell-Smith, Cameron P. Yates, Andrew C. Edwards, Peter J. Whitehead, Brett P. Murphy, Michael J. Lawes

**Affiliations:** 1 Darwin Centre for Bushfires Research, Charles Darwin University, Darwin, Northern Territory, Australia; 2 North Australian Indigenous Land & Sea Management Alliance, Darwin, Northern Territory, Australia; 3 Long Term Ecological Research Network, Australian National University, Canberra, Australia; 4 Research Institute for the Environment and Livelihoods, Charles Darwin University, Darwin, Northern Territory, Australia; University of California Davis, UNITED STATES

## Abstract

Carbon markets afford potentially useful opportunities for supporting socially and environmentally sustainable land management programs but, to date, have been little applied in globally significant fire-prone savanna settings. While fire is intrinsic to regulating the composition, structure and dynamics of savanna systems, in north Australian savannas frequent and extensive late dry season wildfires incur significant environmental, production and social impacts. Here we assess the potential of market-based savanna burning greenhouse gas emissions abatement and allied carbon biosequestration projects to deliver compatible environmental and broader socio-economic benefits in a highly biodiverse north Australian setting.

Drawing on extensive regional ecological knowledge of fire regime effects on fire-vulnerable taxa and communities, we compare three fire regime metrics (seasonal fire frequency, proportion of long-unburnt vegetation, fire patch-size distribution) over a 15-year period for three national parks with an indigenously (Aboriginal) owned and managed market-based emissions abatement enterprise. Our assessment indicates improved fire management outcomes under the emissions abatement program, and mostly little change or declining outcomes on the parks. We attribute improved outcomes and putative biodiversity benefits under the abatement program to enhanced strategic management made possible by the market-based mitigation arrangement.

For these same sites we estimate quanta of carbon credits that could be delivered under realistic enhanced fire management practice, using currently available and developing accredited Australian savanna burning accounting methods. We conclude that, in appropriate situations, market-based savanna burning activities can provide transformative climate change mitigation, ecosystem health, and community benefits in northern Australia, and, despite significant challenges, potentially in other fire-prone savanna settings.

## Introduction

By international standards, Australia’s extensive (~430,000 km^2^) mesic (>1,000 mm.y^-1^) tropical savannas are sparsely populated (<0.1 persons.km^-2^ in rural areas) and little modified since European colonization from the 1850s. Free-range beef cattle production, although often economically marginal, is the dominant land use over ~90% of the region [1,2]. In the absence of intensive land management the region is highly fire-prone. Edwards et al. [3] report that, for the period 2008–2012, contemporary fire regimes over the entire mesic savanna region were characterised by frequent (frequency 0.53 fires y^-1^), relatively severe, extensive fires occurring mostly in the latter part of the 7–8 month (April-November) dry season period.

Although fire is an intrinsic ecological factor regulating the composition, structure and dynamics of savanna systems (e.g. [4–7]), contemporary fire regimes negatively affect community, pastoral production and especially environmental values in northern Australia. Frequent and extensive late dry season fires significantly affect soil erosion and water quality [8–10], fire-vulnerable vegetation [11–13], faunal biodiversity [14–21], and greenhouse gas emissions and related carbon dynamics [22–28].

While our understanding of the magnitude and variety of impacts of contemporary fire regimes on the regional values described above has developed substantially in recent decades, a more formidable challenge lies in implementing cost-effective fire management. This requirement is particularly challenging where fire-vulnerable flora and fauna require patchy fires at small spatial scales (<1 to tens of hectares)—for example, poorly dispersing obligate seeder plant taxa with long maturation periods (>5 years), and relatively immobile vertebrate fauna with small home ranges (<1 –tens of hectares) [18,21,29–31]. Strategic fire management applied intensively over vast landscape scales for delivering appropriate levels of patchiness requires very considerable financial and human resources—well beyond the current means of regional conservation agencies and land (including protected area) managers. Australia's protected areas system depends increasingly on Indigenous Protected Areas (IPAs), whose funding support from government is precarious and insufficient at the best of times [32].

There is significant international interest in the potential alignment of financially incentivised greenhouse gas (GHG) emissions reduction schemes with sustainable environmental outcomes [33–35]. Here we explore the potential for market-based savanna burning GHG emissions abatement and allied carbon biosequestration projects, as means for supporting effective environmental and community co-benefits outcomes in fire-prone savanna environments. Australian experience with nationally recognised savanna burning emissions abatement projects commenced in 2005, substantially in advance of initiatives in other international settings [36].

Globally, savanna fires account for ~60% of contemporary fire emissions [37]—equivalent to ~10–20% of carbon emissions from fossil-fuel combustion in 2011 [38]. Accounting for GHG emissions (specifically CH_4_, N_2_O) and changes in carbon stocks due to savanna fires is a prescribed activity under the Kyoto Protocol [39]. However, the use of fire for climate change mitigation applications is contentious given proscriptive fire management policies in most tropical countries [36,40,41], demonstrated land use and fire impacts on tropical forests [42,43] and savannas [44], and potential deleterious impacts of severe late dry season fire regimes on woody biomass stocks in savannas generally [27,45].

We first contrast fire management outcomes, over a 15-year period, for three major north Australian conservation reserves with an indigenous (Aboriginal) owned and managed, market-based emissions abatement enterprise, the West Arnhem Land Fire Abatement (WALFA) program. While long-term fire regime and biodiversity monitoring data are available for the conservation reserves and show generally deteriorating conditions for fire-vulnerable taxa and communities [46], no similar program nor on-ground assessment data have been reported for WALFA. Hence, we undertake our assessment with respect to three fire regime metrics (fire frequency, long-unburnt vegetation, fire size), each of which is shown to have significant implications for biodiversity management.

Applying nationally accredited savanna burning methodologies, we then assess the carbon credits for each of these properties under realistically achievable, if modest, enhanced fire management. Finally, we consider the ancillary, wider social benefits achieved through the WALFA program, and the relevance of this Australian experience to savanna systems more broadly.

## Methods and Results

### Study sites

**Fig 1:**
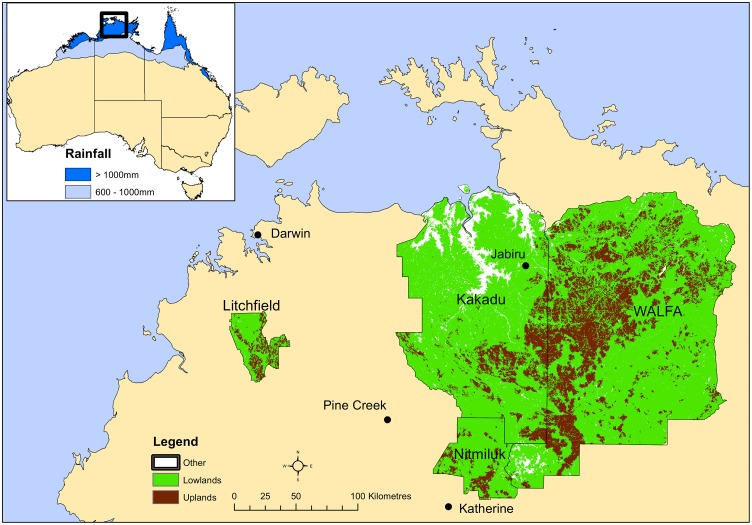
Location of the four study sites in Australia’s ‘Top End’. The inset map depicts two regions for which there are current savanna burning accounting methods—annual rainfall >1,000 mm, and 600–1,000 mm y^-1^. Refer text for details.

Our study sites comprise Kakadu (19,090 km^2^), Litchfield (1,460 km^2^) and Nitmiluk (2,920 km^2^) National Parks, and WALFA (28,000 km^2^), located in the fire-prone ‘Top End’ of the Northern Territory, Australia (Fig 1). The World Heritage Kakadu National Park is co-managed by a national government agency and Aboriginal landowners. Litchfield and Nitmiluk are managed by a Northern Territory government agency, the latter park also under a co-management arrangement with Aboriginal owners.

While varying in size, each of these sites comprises mostly mesic savanna vegetation occurring both in rugged sandstone upland, and undulating laterised sedimentary lowland, settings (Fig 1). Biodiversity values of these savanna systems are recognised as being internationally significant, especially in the rocky uplands [18,47,48].

At all four study sites, prescribed fire management undertaken strategically under mild fire-weather conditions in the early to mid dry season (EDS; generally April-July), aims to reduce the impacts of extensive late dry season (LDS; August-November) wildfires on fire-vulnerable biodiversity. Such EDS fires are, on average, substantially less severe than LDS fires [49,50], more patchy than LDS fires especially in rocky upland terrain [51–53], and yield ~50% less accountable GHG emissions [28]. In large measure, such strategic management practice aims to emulate customary Aboriginal approaches to landscape fire management [54], albeit supplemented with contemporary tools such as ignitions by incendiaries delivered from aircraft and use of global positioning and geographic information systems [55].

The studies reported here were respectively approved and undertaken as part of 20-year formal collaborations at all study sites, initially under the auspices of the Australian Government’s Tropical Savannas Cooperative Research Centre. In more recent years, studies on (1) the three National Parks have been undertaken with Australian Government funding for the Three Parks Savanna Fire Effects Plot Network, a facility of the Long Term Ecological Research Network (LTERN), and (2) for WALFA, since 2006, the formal West Arnhem Fire Management Agreement between the Northern Territory Government and Darwin Liquefied Natural Gas.

### Fire regime metrics

Regional vegetation and fauna are sensitive to frequent, severe and extensive fires (Table 1). In particular, shrubby heathland communities in rocky uplands, characterized by many obligate seeder taxa, are formally listed as an Endangered Community under national legislation [47], and the regional small-mammal fauna is in perilous decline. Contemporary fire regimes are identified as the key threatening process for upland shrublands [47], and a key contributory factor driving the loss of small-mammals [18,21]. Accordingly, we present an assessment of the current efficacy of fire management at each of our four study sites over three successive five-year periods, commencing in 2000, with reference to three fire regime metrics based on current best management practice (Table 2). The first five-year period (2000–2004) pre-dates the formal commencement of the WALFA GHG mitigation project in 2005.

**Table 1:** Published evidence of the sensitivity of plant and animal communities (and component taxa) within the study region to fire regime attributes.

Community and/or taxon	Fire regime sensitivity	Evidence	References
***Vegetation***			
Savanna non-eucalypt shrubs and small trees	Susceptible to intense fires	Unlike the relatively fire-tolerant eucalypts, many other woody savanna taxa are observed to be susceptible in experimental and long-term monitoring studies. Such taxa provide important food and habitat resources for a variety of fauna.	[21,30,92–95]
Old savanna trees, especially those with tree hollows	Susceptible to intense fires	Older trees are observed to be susceptible in experimental and long-term monitoring studies. Trees with hollows provide important nesting and home sites for a variety of birds and small-mammals.	[21,93–95]
Obligate seeder shrubs, especially in upland savannas and heaths	Minimum fire interval of 5 years	Upland savanna species are mostly obligate seeder taxa with 100% mortality from fire; post-fire regeneration from seed only; maturation time for 10% of species ≥ 5 years. Observed declines in obligate-seeder species richness with short fire intervals.	[13,29,96,97]
*Callitris intratropica*—long-lived obligate seeder tree occurring in lowland and upland savannas	Minimum interval of ~10 years between high-intensity fires	Vulnerable to canopy scorching fires; no persistent seedbank; observational studies indicating 10+ years to maturity in typical savanna site conditions.	[11,96,98]
***Fauna***			
Leichhardt’s grasshopper (*Petasida ephippiggera*), in rocky upland sites	Highly susceptible to extensive fires	Spectacular endemic grasshopper with an annual life cycle; restricted to food-plants of the genus *Pityrodia*; immobile nymph populations occurring typically in very small (<<1 ha) local populations.	[99,100]
Bird communities generally—a range of granivorous, frugivorous and hollow-using birds in the tropical savannas	Various species sensitive to frequent, intense and spatially extensive fires	Some granivorous species (e.g. partridge pigeon, *Geohaps smithii*) require a fine-grained mosaic of burnt and unburnt areas. Some species require long-unburnt habitat (e.g. grass wrens, *Amytornis* spp.). Loss of mid-storey trees and shrubs detrimental to frugivorous and insectivorous taxa. Loss of tree hollows affects nesting success of various taxa.	[95,101–104]
Small-mammals—marsupials and rodents, <5.5 kg body mass	Most species sensitive to frequent, intense and spatially extensive fires	Dramatic declines in site-level species richness and individual abundance observed in Kakadu National Park, most pronounced at sites frequently burnt by large (>10 km^2^), homogenous fires. Most small-mammal taxa have home ranges ranging from <1 to tens of hectares. Modelling studies predict long-term declines in a range of species under frequent, high-intensity fires.	[15,19,21,105–107]

**Table 2:** Fire regime metrics and recommended threshold conditions. LDS = late dry season; DBH = diameter at breast height, 1.3 m.

Fire regime metric	Threshold conditions		References
	Lowlands	Uplands	
***Fire frequency***	For small-mammals and many bird taxa, recommended frequency not to exceed 0.3 fires year^-1^	As for lowlands	[76,95]
	(Not applicable)	For persistence of obligate seeder shrub taxa, required fire frequency <0.2 fires year^-1^	[29,96]
	For long-lived obligate seeder tree, *Callitris intratropica*, required frequency of severe / LDS fires <0.2 fires year^-1^ for maintaining tree stems >5cm DBH^a^	As for lowlands	[13]
***Long-unburnt vegetation / habitat***	For small-mammals and birds, 25% of savanna unburnt for at least 3 years	As for lowlands, but 40% be left unburnt for at least three years	[76,95]
	(Not applicable)	For obligate seeder shrub taxa (refer fire frequency above), fire-free intervals of 5+ years required for persistence. Similar issues presumably relate to some vertebrates (e.g. grass wrens, *Amytornis* spp.)	[13,29,104,108]
***Fire size***	For poorly dispersed obligate seeder plants, invertebrates, small-mammals and birds, average patch sizes should be <1 km^2^, preferably less	As for lowlands	[31,95,99,109]
	For small mammal populations, fire size should be <<10km^2^	As for lowlands	[15]

^a^ Derived from modeling presented in [13], where loss of *Callitris* stems is shown to be significantly correlated with the frequency of severe fires (i.e. with mean leaf-scorch heights >2 m). For present purposes, we assume that frequency of severe fires is broadly relatable to the frequency of LDS fires given that the probability of any LDS fire being severe is 0.81 [13].

For all fire regime assessments presented below, and as applied to GHG emissions abatement and carbon sequestration calculations in the following section, fire mapping data were derived from MODIS (Moderate Resolution Imaging Spectroradiometer) satellite imagery (250 m resolution) using procedures and validation methods described by Fisher and Edwards [56]. This imagery has been available globally from 2000. Methods used for undertaking respective fire regime metric assessments are provided as Supplementary Information in S1 Appendix. GIS files delineating Upland and Lowland landscape units are provided for the four study sites respectively as spatial layers zipped in Supplementary files S1 File. GIS files for Kakadu Landscape Units, S2 File. GIS files for Litchfield Landscape Units, S3 File. GIS files for Nitmiluk Landscape Units, S4 File. GIS files for WALFA Landscape Units.

### Fire frequency

**Fig 2:**
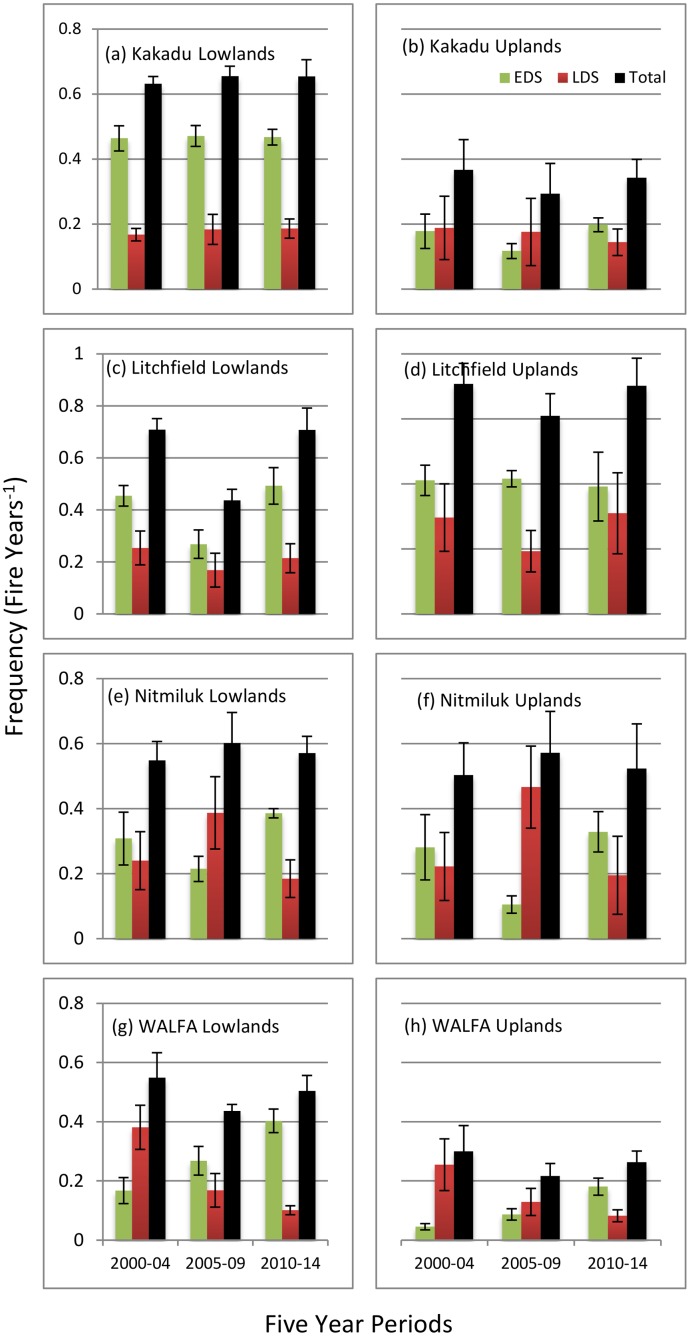
Mean frequency (± S.E.M.) of early dry season (EDS—pre-August), late dry season (LDS—post-July), and total (annual) fires in three successive five-year periods (commencing 2000), for the four study sites and respective lowland and upland savanna units.

For lowland and upland units at the four sites the mean frequency of EDS and LDS fires was calculated in three successive five-year periods (Fig 2). In the five-year period prior to the implementation of strategic fire management in WALFA, the fire regime was dominated by LDS wildfires; in subsequent periods the influence of EDS strategic fire management has been more pronounced, especially in lowland settings (Fig 2g and 2h). For Kakadu (est. 1979), a similar shift was observed in the seasonal patterning of burning from the mid-1980s once a more strategic approach to fire management was implemented [57].

While the recommended annual fire frequency to conserve vertebrate fauna should not exceed one year in three for savanna habitats (Table 2), this threshold is substantially exceeded for lowland habitats at all study sites, and (notably given particular fire sensitivity of sandstone heaths) at Litchfield and Nitmiluk in upland habitats (Fig 2).

For obligate seeder shrub taxa occurring in rocky upland habitats susceptible to fires at frequencies >0.2 y^-1^, this threshold has been exceeded over the entire study period at all study sites, but most strikingly in Litchfield and Nitmiluk (Fig 2).

For the long-lived obligate seeder tree, *Callitris intratopica*, which is susceptible to relatively severe LDS fires at frequencies >0.2 y^-1^, this threshold has been achieved over the entire study period in Kakadu, since the implementation of the strategic management program in WALFA, and in some periods in Litchfield and Nitmiluk (Fig 2).

### Long-unburnt vegetation

**Fig 3:**
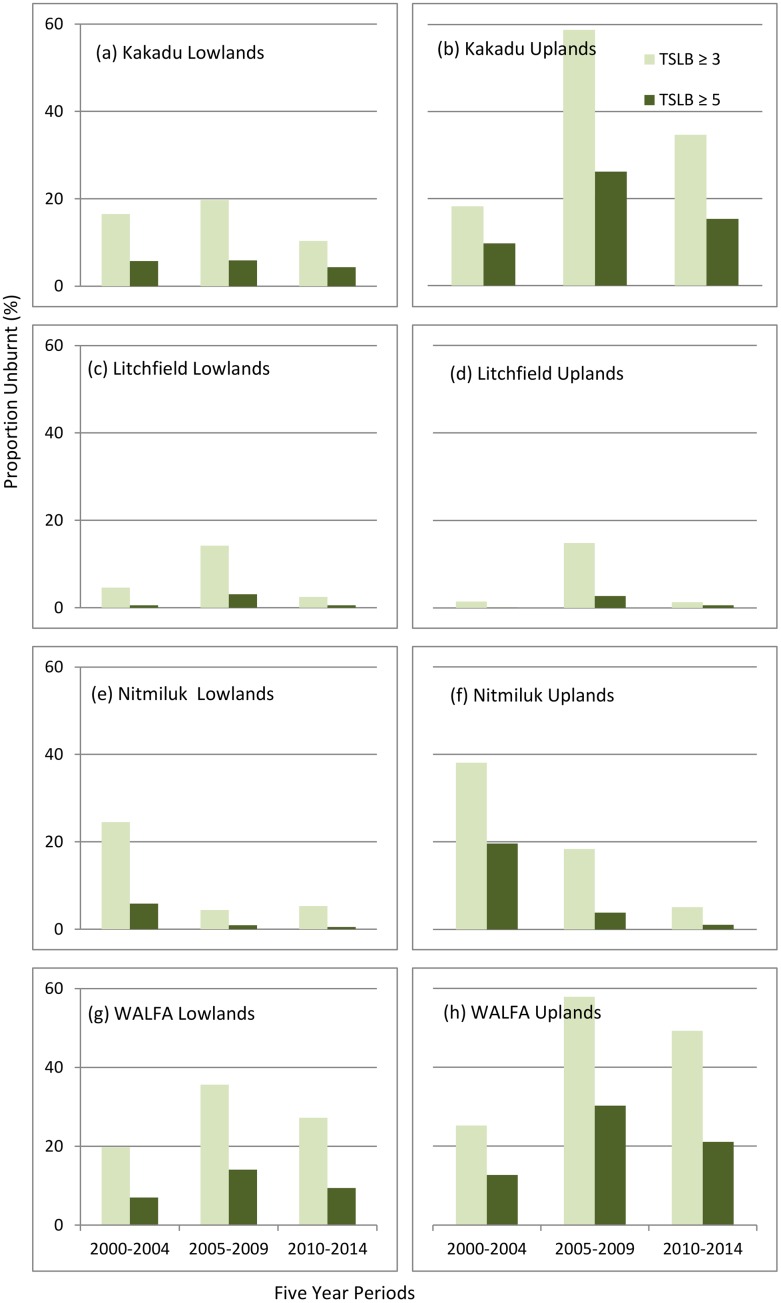
Long-unburnt vegetation, expressed as the proportion of each study site remaining unburnt (for ≥3 and ≥5 years) at the end of respective five-year periods, for lowland and upland savanna units. TSLB = time since last burnt.

Over the 15-year assessment period there has been slight improvement in the proportion of lowland habitat unburnt for at least 3 or 5 years in WALFA, and relatively substantial improvement in the proportions of similarly unburnt upland habitat both in WALFA and Kakadu (Fig 3). By the third assessment period, only WALFA exceeded recommended thresholds (Table 2) of 25% of lowlands, and 40% of uplands, remaining unburnt for at least 3 years. The situation with respect to these recommended thresholds in Litchfield and Nitmiluk is evidently parlous, and at Nitmiluk there has been a precipitous decline in the proportion of unburnt habitat since the first assessment period (Fig 3).

The apparent contradiction in the observed proportions of lowland habitat frequently burnt in WALFA (exceeding 0.4 fires year^-1^ in each assessment period, Fig 2) versus >25% remaining unburnt in the latter two assessment periods (Fig 3), reflects application of relatively frequent prescribed burning focused on strategic locations (e.g. reinforcing natural fire-break barriers such as tracks and watercourses) with the effect of increasing the proportion of unburnt habitat in WALFA lowland habitats generally.

### Fire patch-size

#### Average fire patch-sizes

The mean annual patch-sizes of contiguously burnt areas (CBAs) and counts of the number of CBAs, over successive five-year assessment periods, are presented in Table 3a and 3b respectively. CBA distributions were calculated separately for EDS, LDS and annual periods, and comprise both orthogonally and diagonally contiguous burnt pixels. Mapped CBAs may thus comprise a number of individual fires in any one fire season, and annual CBAs will likely include (a) EDS fires that continued to burn into the LDS, and (b) separate EDS and LDS fires that coalesced.

**Table 3:** Contiguous burnt area (fire patch-size) characteristics (± S.E.M.) for study sites over three assessment periods, (a) average fire patch-size, (b) count of patches. EDS = early dry season; LDS = late dry season.

Site	Assessment period								
	2000–2004			2005–2009			2010–2014		
	EDS	LDS	Annual	EDS	LDS	Annual	EDS	LDS	Annual
***(a) Patch size (km*** ^***2***^ ***)***									
**Kakadu**	23.7 ±7.1	16.0 ±2.8	47.0 ±6.6	15.1 ±2.8	11.1 ±2.1	34.2 ±6.6	20.6 ±2.3	13.8 ±2.5	49.9 ±6.6
**Litchfield**	24.5 ±4.6	51.6 ±34.2	73.1 ±33.4	14.9 ±2.8	22.1 ± 5.0	35.6 ±32.6	22.3 ±9.3	33.4 ±13.1	63.0 ±33.6
**Nitmiluk**	30.2 ±18.9	43.2 ±18.7	99.8 ±19.8	9.7 ±3.9	65.9 ±21.2	97.3 ±20.3	25.5 ±10.1	29.9 ±11.8	75.3 ±18.1
**WALFA**	17.2 ±5.9	51.9 ±10.3	86.8 ±12.3	7.7 ±1.8	14.5 ±3.2	17.0 ±14.8	16.0 ±2.9	10.1 ±2.2	28.7 ±14.7
***(b) Count of patches***									
**Kakadu**	378 ±81	204 ±20	249±47	506 ±64	286 ±58	341 ±56	356 ±21	240 ±26	239 ±36
**Litchfield**	28 ±3.9	17 ±5.6	19±3.5	45 ±5.3	13 ±1.3	29 ±5.1	38 ±6.5	13 ±1.5	21 ±4.6
**Nitmiluk**	47 ±9.5	15 ±2.5	31±11.9	55 ±11.3	22 ±2.3	34 ±13.8	53 ±9.6	15 ±3.6	39 ±10.5
**WALFA**	266 ±68	181 ±16	195±60	730 ±63	298 ±58	604 ±72	578 ±50	305 ±70	464 ±68

Average fire-patch sizes were substantially larger than recommended thresholds (<1 km^2^, Table 2) at all study sites (Table 3a). While this result is largely an artifact of the relatively coarse sensor-scale (MODIS) used for fire mapping, of note is (1) the substantial reduction in both average LDS and annual fire patch-sizes in WALFA, and (2) in the third assessment period, the generally larger EDS and LDS fire patch-sizes, and substantially larger annual fire patch-sizes, at national park sites (Table 3a). Concomitant improvement in implementation of a more strategic fire management program in WALFA is reflected in the mean number of prescribed EDS CBAs rising from 266 in the first pre-project period, to 730 and 578 respectively in subsequent periods (Table 3b).

#### Fire patch-size distributions

**Fig 4:**
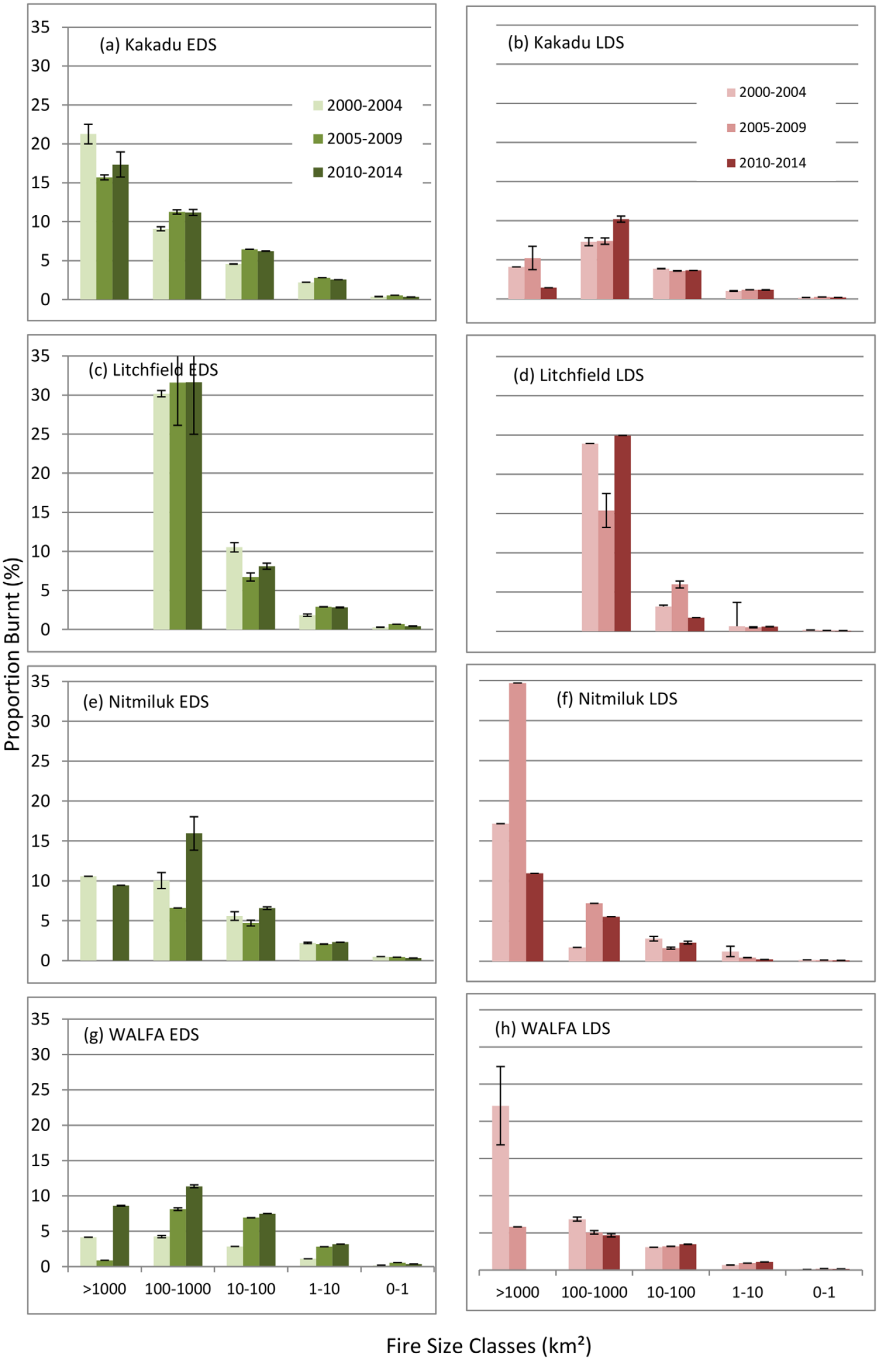
Mean early dry season (EDS) and late dry season (LDS) fire patch-size distributions (± S.E.M.) of contiguously burnt areas (CBAs) over successive five-year periods for the four study sites, expressed as the proportion of respective lowland and upland savanna units.

The mean annual patch-size distributions of CBAs over successive five-year periods, expressed as the proportion of respective study sites affected by fire, were examined separately for EDS and LDS periods (Fig 4). For WALFA, there was a substantial reduction in the contribution of the largest (>1000 km^2^) CBA class since 2005 (Fig 4), and general, albeit slight, reduction in the proportion burnt in CBA classes >10 km^2^ over the three assessment periods (respectively: 43%, 30%, 36%). This latter trend was associated particularly with the reduced contribution of relatively severe LDS fires >10km^2^, from 42% in the first assessment period to 8% in the third.

For the three National Parks, the contributions of CBAs >10km^2^ have shown no improvement over the three assessment periods (Fig 4): Kakadu increasing from 45% to 50%; Litchfield, varying from 61% to 70%; Nitmiluk, varying from 48% to 53%. Little change is evident in the contributions of LDS fires to CBAs >10km^2^ over the three assessment periods for Kakadu (16–17%) and Litchfield (21–27%), with more erratic performance in Nitmiluk.

## Discussion

### Fire regime metrics assessments

Although contemporary north Australian fire regimes are generally environmentally unsustainable, periodic fire disturbance is an essential factor regulating regional savanna systems. However, given that most regional fires are of anthropogenic origin [58] and occur most extensively as LDS wildfires [3,58], a key challenge is to implement strategic prescribed fire management under mild fire-weather conditions in the EDS. The development of contemporary north Australian fire regimes dominated by frequent LDS fires is attributable to the cessation of widespread fine-scale indigenous fire management practice from the late 19^th^ century onwards following European colonization [11,59–62].

While our current understanding of fire ecology is imperfect (particularly in relation to understanding interacting effects of fire frequency and patch-size distributions on relatively immobile fauna [15,30,31,63] sufficient information is available to broadly describe ecologically appropriate fire regime thresholds (Table 2), as well as to inform practical methodologies for delivering them [54,64,65].

Our assessment indicates that, overall, there has been general improvement in the implementation of fire management in WALFA once the mitigation program commenced after the first assessment period—most notably through a marked change in the season of burning to generally less severe EDS burns (Fig 2), an increased proportion of unburnt vegetation or habitat (Fig 3), and a substantial decline in the incidence of very large (>1000 km^2^) relatively severe LDS fires (Fig 4). In the parks, there has been notable improvement in the proportion of long unburnt vegetation in the Kakadu uplands—but otherwise there has been no improvement in most metrics, and a precipitous decline in the proportion of long unburnt vegetation in Nitmiluk.

By the third assessment period, substantial fire management challenges still remain, particularly with: (a) the very high frequency (~0.6 fires y^-1^ or greater) of burning and consequently little unburnt (≥3 y) vegetation remaining (~10% or less) in Kakadu lowlands, and in Litchfield and Nitmiluk generally; and (b) the ongoing contribution of large (>10km^2^) fires at all sites, ranging from an annual mean of 36% of the entire WALFA area, ~50% of both Kakadu and Nitmiluk, to 67% of Litchfield.

The fire patch-sizes of CBAs reported here are largely an artifact of the imagery scale used for mapping—especially the omission of large numbers of smaller fires (<10km^2^) when mapping is derived from relatively coarse resolution imagery (e.g. MODIS as used here, or AVHRR [Advanced Very High Resolution Radiometer, 1.1 km pixels]). The latter can be illustrated by examining validated fire mapping derived mostly from Landsat MSS (79 x 57 m pixels) and Landsat ETM and TM imagery (25 m pixels) for a slightly reduced WALFA area than assessed here. Over a 9-year period (1997–2005), Yates et al. [31] mapped an annual average of ~13,200 CBAs of which 99.5% were <10 km^2^, with an average fire patch-size of 1.2 km^2^ in the EDS and 4 km^2^ in the LDS. By contrast, based on fire mapping for a 630,000 km^2^ savanna region (including the WALFA sample) derived from AVHRR imagery over the same time period, Yates et al. [31] mapped an annual average of ~1,800 CBAs, with an average fire patch-size of 35 km^2^ in the EDS and 185 km^2^ in the LDS. Importantly, however, those authors [31] observed that, at both imagery scales, very large (>1,000 km^2^) fires, while few in number, contributed a significant proportion (AVHRR—65%, Landsat—82%) of the mean annual total area affected by fire.

Prior to the commencement of the WALFA program in 2005, from 1990 to 2004 an average of 36.5% of the project area was burnt each year, of which 73% comprised LDS wildfires [66]. During this period little concerted fire management was undertaken, resulting in a boom-and-bust cycle of landscape-scale fuel accumulation followed by extensive LDS wildfires occurring approximately every 3 years [51]. In the subsequent seven years, an annual average of ~32% of WALFA was burnt, of which two thirds comprised EDS prescribed fires [55]. This improvement occurred under regionally deteriorating fire-weather conditions, especially the increased number of hot days >37°C [67].

The generally improved fire management program in the WALFA region, and the putative biodiversity benefits associated with that improvement (Tables 1 and 2), could only have been achieved through the implementation of an effective and relatively well funded GHG emissions mitigation program.

### Fire management and the carbon economy

Australia’s National Greenhouse Gas Inventory (NGGI) has recognised GHG emissions offsets generated through savanna burning projects under high rainfall conditions (mean annual >1,000 mm) since 2012, based on the method formally described and regulated in the Carbon Farming Methodology of 2012 [68]. An essential principle of that method is that it accounts for emissions abated in any one year against a 10-year pre-project baseline, through application of strategic fire management to reduce the impacts of, and generally greater emissions from, extensive and severe wildfires [55].

A slightly amended version of the Carbon Farming Methodology has been released [69] which will underpin new savanna burning projects to be delivered as part of the Australian government’s Emissions Reduction Fund (ERF) arrangements. The ERF aims to reduce Australia’s GHG emissions by 5% relative to 2000 levels by 2020, through public purchase of credits generated using endorsed methods (see [69]), including the current iteration of the savanna burning emissions abatement method. A key element of the ERF is that projects securing contracts through a reverse auction process (i.e. the lowest bids are accepted) will have to deliver carbon credits for up to seven years. The first ERF auction in April 2015 returned a price of AU$13.95 per tonne CO_2_-equivalent. Projects will also be able to sell carbon credits into voluntary markets (e.g. corporates seeking social-license-to-operate; national sovereign funds; and international not-for-profit carbon credit brokers) that recognise approved Carbon Farming Methodologies, including participants in Australia's Carbon Neutral Program.

Under ERF rules, savanna burning GHG offset projects are permitted on all tenure types, including conservation reserves, provided there is no existing regulatory requirement (such as in a formal Plan of Management) for fire management objectives to include reducing GHG emissions or sequestering carbon. These arrangements thus afford novel opportunities for addressing fire and biodiversity management imperatives on the fire-prone savanna conservation estate.

Based on fire management experience with WALFA, we have calculated the quantum of carbon credits which, realistically, could be generated annually at all four study sites with respect to three savanna burning methods (see S1 Appendix). Given the poor condition generally of current fire management in each of our national park study sites (Figs 2–4), we have assumed that these properties could achieve at least the level of abatement (38% reduction in emissions) achieved in WALFA’s first seven years of operation [55].

The first of those methods concerns the recently revised abatement method [69], which also extends the savanna climate envelope to the 600 mm mean annual rainfall isohyet (Fig 1). The second is a novel method to account for carbon sequestration into fine and coarse woody fuels [22] currently being finalised, with approval for implementation due in 2016. The third is a method under advanced development addressing sequestration in living tree biomass based on statistical modeling of an extensive plot-based dataset [23,27]. All three methods are complementary (i.e. additive) in that they are reliant on the same enhanced fire management activity but account for different GHG and carbon pool components. Calculation procedures are given in S1 Appendix.

The magnitude of opportunities, and associated statistical uncertainties, afforded by the different methods to generate carbon credits for the four study sites are given in Table 4. Carbon market opportunities are especially prospective in extensive fire-prone lowland savannas, and for sequestration components. WALFA has achieved the annual level of GHG emissions abatement indicated, based on a switch to a more strategic EDS fire management program, resultant lower severity fires, and reduction in burnt area overall [55]. Given the relatively large proportions of especially lowland savannas burnt annually in the three national parks, we see no reason why equivalent emissions abatement and sequestration benefits cannot be achieved with more strategic management. Based on current Australian experience, the prospective value of generated credits under ERF arrangements can be anticipated to exceed US$10 per tonne of CO_2_-e.

**Table 4:** Indicative carbon credits feasibly generated using three complementary savanna burning methodologies under enhanced fire management. (1) each t.CO_2_-e.y^-1^ abated or sequestered = 1 carbon credit; (2) enhanced fire management scenarios based on published experience with WALFA [55]; FCWF = fine and coarse woody fuels; (3) for sequestration methods, carbon credits are annualized over 25 years. (4) Brief footnotes concerning method uncertainties are provided below; other details concerning respective methods, calculations, and management assumptions are presented in Supporting Information.

Site	Landscape unit	Area (ha)	Indicative carbon credits (X 10^3^)			
			Method			Total
			*GHG emissions*	*FCWF biomass*	*Living tree*	
			*abatement* ^a^	*sequestration* ^b^	*biomass sequestration* ^c^	
			(t.CO_2_-e.y^-1^)	(t.CO_2_-e.y^-1^)	(t.CO_2_-e.y^-1^)	(t.CO_2_-e.y^-1^)
**Kakadu**	Lowland	1,506,144	78	338	474	886
	Upland	98,538	3.3	0	30	33.3
**Litchfield**	Lowland	106,881	6.4	29	34	69.4
	Upland	20,506	1.2	1.3	6.2	8.7
**Nitmiluk**	Lowland	246,231	15	59	77	151
	Upland	23,856	1.4	0.4	7.2	9
**WALFA**	Lowland	2,055,131	121	343	603	1,067
	Upland	392,650	19	26	115	160

^a^ The original emissions abatement methodology incorporated a formal uncertainty assessment for the WALFA region [28], where domain emissions were found to be accurate at the 95% confidence level to within a factor of 30–35% of the mean, with an overall coefficient of variation = 0.16.

^b^ Key steady state fuel load parameters for the FCWF biomass sequestration methodology [22] are derived directly from the original emissions abatement methodology, which includes formal assessment of individual parameter uncertainties [24]. The method is also reliant on reliable fire mapping derived from MODIS imagery, estimated as being 88% accurate when compared with field data [56].

^c^ The living tree biomass sequestration methodology [22] expands on work reported in [27], where the best linear mixed effects model (p < 0.005; R^2^ = 0.11, determined using Akaike’s Information Criterion), assessing the effects of 7 variables (annual rainfall; plot basal area; fire severity variables, fire seasonality variables) on stem increment, incorporated only fire severity terms. Data were derived from 135 40 X 20 m plots over 10 years of observations. Work on this methodology is ongoing, especially to address potential effects of severe fires on bark removal with implications for calculation of biomass using allometric relationships [23].

Despite this optimistic outlook considerable hurdles lie ahead, especially for biosequestration projects which face fluid policy uncertainties, including (1) significant untested land tenure, carbon property rights, governance and regulatory issues, and (2) definitions of permanency (i.e. maintaining carbon benefits for multi-decadal periods) [55]. For instance, the ERF allows for 7-year contracts but requires that permanency be maintained for 100 years with a discounted option for 25 years [70]. Biosequestration projects are thus likely to be considered only in situations where tenure is assured and in the absence of other viable land use options. In north Australia, these conditions pertain to extensive valuable conservation estate, especially on indigenous-owned lands, including national parks.

### Delivering multiple benefits

As demonstrated by WALFA, enhanced fire management undertaken for the purposes of GHG emissions abatement and carbon sequestration can be compatible with delivering biodiversity conservation outcomes in north Australian mesic savannas. Important exceptions include situations where alternative emissions-intensive fire management approaches are required; for example, addressing woody thickening and encroachment affecting pastoral production and conservation values [71]; and management of high biomass flammable exotic grass species [72,73]. Notably, Australia’s current and developing savanna burning methodologies (Table 4) apply only to wooded savannas with foliage projective cover >10%.

Enhanced savanna fire management, focusing especially on the reduction of current severe LDS fire regime impacts, can deliver substantial benefits to ecosystem health including biomass sequestration and fire–vulnerable biodiversity, soil erosion and stream sediment transport [3], and reduced airborne particulates using the same management practices that reduce greenhouse gas emissions [74]. In the absence of reliable and adequate private and/or public funding to address all of these issues directly, carbon and broader ecosystem services markets provide an obvious solution.

The resourcing available to the three state-owned reserves to deliver effective fire management and monitor biodiversity outcomes has been in substantial decline over the past decade, consistent with the ongoing trend for protected areas nationally [75]. The consequences of this declining commitment are reflected in the current trajectory of the population status of a variety of taxa, including many formally listed threatened taxa [47,76]. Against this trend, fire management in Kakadu’s rugged biodiverse uplands has been generally more effective over the life of the park, including concerted strategic management in recent years [77].

Although biodiversity impacts are apparent also in the WALFA project region [47], from 2006 significant enhancements to the delivery of fire management (Figs 2–4) have been made as a direct result of both: (1) substantially increased funding, associated with the engagement of local indigenous communities and individuals in delivering the program (including employment of >200 individuals in any one year); and (2) building the institutional capacity of local management organisations. That performance has been made possible through short-term (<3–5 year) Australian Government funding programs for indigenous ranger positions and operational activities, critically underpinned by a longer-term (17-year) commercial savanna burning emissions abatement contract with a global energy corporation [55].

The adequacy and sustainability of funding has particular resonance for Australia’s Indigenous Protected Area (IPA) program which, in the next few years, is anticipated to contribute ~50% of Australia’s National Reserve System, including substantial areas of north Australia. Funding of IPAs is not guaranteed beyond 2018 and, based on 2012–13 budget figures, is estimated to be an order of magnitude less (per hectare) than that available for state-run reserves [32]. Much of WALFA is already included in IPAs.

The singular importance of supporting the capacity of indigenous land owners and residents to be part of the regional conservation solution is underscored by observations that Aboriginal people constitute the majority of the remote north Australian rural population, are financially impoverished despite being land-rich, and possess recognised skills in, and cultural responsibilities for, landscape fire management [78]. By stark contrast with WALFA, the lack of committed indigenous engagement and resultant disillusionment is identified as a key issue underlying the recent history of fire management in Kakadu [36,79].

The examples of WALFA and emerging projects at other north Australian savanna sites, coupled with substantial additional opportunities identified in Table 4, show that incomes from carbon markets offer realisable transformative benefits for climate change mitigation, biodiversity conservation, and community well-being and social development across the fire-prone savannas of north Australia.

### Application in other savanna settings

Considerable if untested potential exists for application of savanna burning abatement and sequestration methodologies supporting biodiversity conservation and livelihood outcomes in fire-prone savannas in southern Africa, Asia, and South America [36]. As well as the Australian examples described here, an accredited savanna fire management methodology for assessing carbon stocks recently has been approved for application in east African miombo woodlands [80].

However, implementation challenges are substantial and context-specific, for example: adapting to site and project-scale social, ecological, and technical drivers; capability development and investment requirements; accounting for complex national tenure, regulatory, and governance arrangements [33,36,81]. Across sub-Saharan Africa, Southeast Asia, and South America, most national regulatory forestry and fire management frameworks either prohibit fire use or, at best, stipulate only fire prevention and suppression actions [36]. As demonstrated by ongoing high incidence of uncontrolled fires in these continental settings, such policies have both failed and, worse, discouraged sustainable fire management practices associated with many traditional agricultural, or community-based, activities [40,41].

An illustrative example of such policy failure is afforded by Southeast Asia where, despite lack of reliable mapping of the distribution of savanna (including derived savanna) vegetation [82], as many as 30 million people use fire as part of traditional agricultural and agroforestry practice in highly fragmented, densely populated landscapes [83–85]. While the official policy of the Association of South East Asian Nations (ASEAN) since 1999 is to actively discourage traditional practices incorporating fire applications, this policy reflects a long history of lack of understanding of the practical livelihood and environmental benefits that traditional agro-forestry systems can deliver and denial of major causes of regional fire management problems attributable mostly to deforestation and degradation associated with poor forestry and commercial-scale agricultural management practices [84,86]. A general assessment of the applicability of and challenges associated with savanna burning projects in fire-prone southern African (especially Namibia) and South American (especially Venezuela) settings is provided in Russell-Smith et al. [36].

For biosequestration projects especially, insecure (typically traditional communal) tenure is problematic for indigenous peoples and rural communities in many international contexts [81,87] where multi-decadal ‘permanency’ provisions need to be assured [88]. For marketability, savanna fire management methodologies also need to meet strict accreditation requirements (including monitoring and verification) under various international protocols (e.g. Clean Development Mechanism, REDD+), or accepted voluntary standards (e.g. Voluntary Carbon Standard, Gold Standard).

Furthermore, the spatial scales at which savanna burning projects are conducted in north Australia are unlikely to be replicable in many other international settings—for example, fire-prone savanna regions throughout Asia and many parts of Africa and the Americas typically are relatively densely populated and highly fragmented. While direct financial benefits attributable to emissions reductions through savanna burning may be commensurately small, nonetheless encouraging more sustainable forms of savanna fire management that complement other sustainable agricultural practices at larger (for example catchment) scales, can contribute substantially to enhanced livelihood benefits [89].

In contrast to extensive experience with GHG mitigation activities in tropical forests (e.g. through REDD+), to date there has been little opportunity to apply analogous market incentives to help conserve fire-prone savannas—despite much wooded savanna falling within the accepted broad definition of forest (e.g. [90]) and being subject to rates of conversion possibly twice that of tropical forests [91]. Despite evident challenges, given the magnitude of pressing environmental and livelihood issues in savanna systems globally, mitigation programs rewarding enhanced savanna fire management have the potential to offer significant multiple benefits.

## Supporting Information

S1 Appendix Calculating fire metrics and carbon credits. (DOCX)

S1 File GIS files for Kakadu Landscape Units. (ZIP)

S2 File GIS files for Litchfield Landscape Units. (ZIP)

S3 File GIS files for Nitmiluk Landscape Units. (ZIP)

S4 File GIS files for WALFA Landscape Units. (ZIP)
