# Supplementary material for: Deriving Multiple Benefits from Carbon Market-Based Savanna Fire Management: An Australian Example
Source: PLoS One. 2015 Dec 2;10(12):e0143426. doi: 10.1371/journal.pone.0143426 (PMC4668068; doi:10.1371/journal.pone.0143426)
Supplement: S1 Appendix — (DOCX) [file pone.0143426.s001.docx]

**S1 Appendix. Calculating fire metrics and carbon credits**

**1. Calculating fire regime metrics**

For all fire regime metric assessments, fire mapping data were derived from MODIS (Moderate Resolution Imaging Spectroradiometer) imagery (250 m resolution) using procedures, and detailed validation assessments, as described in [[1](#_ENREF_1)]. This fire history dates back to 2000. Fire mapping layers are annual (by calendar year), attributed by month and publicly available on the North Australia Fire Information (NAFI) website (<http://www.firenorth.org.au/nafi3/>). All spatial analyses were undertaken as raster in ArcGIS [[2](#_ENREF_2)] and ArcInfo workstation [[3](#_ENREF_3)].

Following convention in north Australian fire ecological studies describing fire seasonality [[1](#_ENREF_1)], for all assessments presented here the early dry season (EDS) describes burning occurring in the period Jan – Jul, and the late dry season (LDS) describes the period Aug – Dec.

For each of the four study sites ( Kakadu, Litchfield, Nitmiluk National Parks; and the West Arnhem Land Fire Abatement [WALFA] project area) assessments were undertaken with respect to ‘lowland’ and ‘upland’ savanna landscape units. Respective lowland and upland surfaces were derived from independent mapping of ‘eligible fuel types’ available from the SavBAT emissions calculator tool on the NAFI website. For assessment purposes, our ‘lowland’ unit combines mapping for Open-forest mixed and Woodland mixed fuel types. Our ‘upland’ unit uses mapping only for the “Woodland with hummock grass understorey” fuel type, and does not use mapping for more specialised, essentially non-savanna, upland vegetation described as the “Shrubland with hummock grass understorey” fuel type (also referred to as Sandstone Heath). The same derived mapping surfaces describing these ‘lowland’ and ‘upland’ units were used for carbon credit calculations described in the section following.

Using these mapping data, three fire regime metrics were derived using standard GIS procedures, as follows:

*Fire Frequency*—the mean frequency of EDS and LDS fires, and the annual TOTAL, were calculated for three successive five-year periods (2000-04, 2005-09, 2010-14), for lowland and upland units in each of the four study sites.

*Long-Unburnt—* for respective 5-year assessment periods, annual fire mapping data attributed as burnt were overlaid in descending yearly order. That is, for any given assessment period, the top or most recent year was attributed with ‘one’, the next year with ‘two’, and so on. Fire-free interval distributions, where each year unit describes the proportional burnt extent of respective lowland and upland units, were then derived for 5-year periods. Reported values of ≥3, and ≥5, fire-free intervals were derived from these data.

*Fire size (contiguously burnt areas [CBAs])*—fire patch sizes, comprising both orthogonally and diagonally adjacent burnt pixels, were determined separately for EDS and LDS periods for respective years over the 15 year study period. CBAs were grouped into 5 size classes (0-1 km^2^; 1-10 km^2^, 10-100 km^2^, 100-1000 km^2^, >1000 km^2^). The mean proportion of lowland and upland units burnt by fires in respective CBA classes was then calculated for each site in respective five-year assessment periods.

**2. Calculating carbon credits for the four study sites using three savanna burning accounting methods**

The quantum of carbon credits (t CO_2_-e) which could be generated annually for lowland and upland units at the four study sites is calculated here with respect to three complementary savanna burning methods: (1) the latest approved version of the abatement methodology [[4](#_ENREF_4)]; (2) sequestration in non-living, fine and coarse fuel biomass [[5](#_ENREF_5)]; and (3) sequestration by living tree biomass, based on statistical modelling of 20 years of plot data (*n*=134) [[6](#_ENREF_6)] [[7](#_ENREF_7)]. All three methods are complementary (i.e. additive) in that they are reliant on the same enhanced fire management activity but account for different greenhouse gas and carbon pool components.

To achieve any carbon credits, each of these methods requires that the abatement, or sequestration, is measured against a baseline (or pre-project) scenario. All three methods require a 10-year baseline. For our calculations, we have assumed that improved fire management (IFM) in the three national parks could achieve the same relative level of enhancement as that achieved by the Western Arnhem Land Fire Abatement (WALFA) project. Contemporary indigenous fire management practices re-introduced in WALFA in 2005, resulted in an 8% reduction in overall fire frequency and a substantial increase in the EDS/LDS ratio from 0.25 to 1.9 (8% and 32%, to 21% and 11%) for 1995-2004 and 2005-11, respectively [[8](#_ENREF_8)].

For WALFA, the baseline period is 1995-2004, and the project period 2005-2014 (Table S1). The baseline period for the national parks is 2005-2014.

**Table S1: Proportions of respective study sites burnt in baseline periods and, for WALFA, in the project period.** EDS = early dry season (pre-August), LDS = late dry season.

| **Site** | **Period** | **Unit** | **Proportion of site burnt** | | |
| --- | --- | --- | --- | --- | --- |
|  |  |  | ***EDS*** | ***LDS*** | ***Annual*** |
| WALFA | Baseline (1995-2004) | Lowland | 0.17 | 0.37 | 0.54 |
| WALFA |  | Upland | 0.07 | 0.29 | 0.36 |
| WALFA | Project (2005-2014) | Lowland | 0.29 | 0.16 | 0.45 |
| WALFA |  | Upland | 0.11 | 0.10 | 0.21 |
|  |  |  |  |  |  |
| Kakadu | Baseline (2005-2014) | Lowland | 0.41 | 0.18 | 0.60 |
| Kakadu | Baseline (2005-2014) | Upland | 0.18 | 0.14 | 0.32 |
|  |  |  |  |  |  |
| Litchfield | Baseline (2005-2014) | Lowland | 0.43 | 0.24 | 0.66 |
| Litchfield | Baseline (2005-2014) | Upland | 0.36 | 0.27 | 0.64 |
|  |  |  |  |  |  |
| Nitmiluk | Baseline (2005-2014) | Lowland | 0.25 | 0.32 | 0.57 |
| Nitmiluk | Baseline (2005-2014) | Upland | 0.20 | 0.31 | 0.51 |

Details of the accounting methods are described below. We note that (1) all three methods are reliant on knowing the seasonal proportions of vegetation fuel types burnt, and (2) the abatement and living tree biomass sequestration methods also apply seasonal probability constants for the proportions of low, moderate and high severity fires for respective vegetation fuel types, following [[9](#_ENREF_9)].

**Savanna burning methods**

*Emissions abatement*

When we refer to greenhouse gas emissions abatement, we refer specifically to the abatement of the gases methane and nitrous oxide [[4](#_ENREF_4), [10](#_ENREF_10)]. Greenhouse gas emissions abatement was calculated using the on-line Savanna Burning Abatement Tool, version 2 (SAVBat2, [www.savbat2.net.au](http://www.savbat2.net.au)), which applies an updated version of the savanna burning accounting method [[4](#_ENREF_4)]. The user uploads a GIS vector boundary for nominated project sites, and SAVBat2 uses pre-defined Vegetation Fuel Types (VFTs) mapping [[11](#_ENREF_11)] to calculate available fuel loads. To estimate methane and nitrous oxide emissions, SAVBat2 uses the burnt area mapping product available from NAFI.

A report is generated by SAVBat2 describing greenhouse gas emissions for each year stipulated, for the four VFT classes. For present purposes *Open Forest* and *Woodland Mixed* VFTs were combined to calculate lowland emissions, and *Woodland Hummock* was used for calculating upland emissions. We do not present calculations for the relatively infrequently burnt *Shrubland Hummock* VFT.

Baseline emissions for lowland and upland units in the three national parks were calculated as mean annual emissions over respective 2005-14 periods, and correspondingly for WALFA for the 1995-2004 baseline period. Mean annual emissions under IFM for WALFA were calculated for the period 2005-2014. The potential for emissions reduction for lowland and upland units in the respective national parks was calculated as the mean baseline emissions per unit area (t CO_2_-e ha^-1^) minus the corresponding mean IFM emissions abatement per unit area achieved for WALFA.

*Carbon sequestration in fine and coarse fuel*

Carbon sequestration in fine and coarse woody fuel (FCWF) was calculated using the method outlined by [[6](#_ENREF_6)]. The areal proportions of respective VFT classes burnt annually (*Frq*) and in the EDS (*P_E_*), averaged for the baseline period, are used as inputs for calculating the total FCWF biomass using Eqns 1-4 in [[6](#_ENREF_6)]. Using inputs of *Frq* and *P_E_* those same equations and Eqn 5 are used to calculate total C sequestered (t CO_2_-e ha^-1^) over a subsequent 25-year, with-project period. The potential sequestration in fine and coarse fuel (Seq_T_) is the product of (Eqn 1): the difference in the baseline (S_Tb_) and the value calculated for the WALFA IFM period (S_Tp_); the total area of the VFT; and 0.95, which allows for a 5% buffer as per Emissions Reduction Fund requirements:

Seq_T_ = (S_Tb_ - S_Tp_) * Area_(VFT)_ * 0.95 (Eqn 1)

*Carbon sequestration in above-ground tree biomass*

Cook et al*.* [[6](#_ENREF_6)] developed a statistical model of annual change in above-ground tree biomass carbon stock in response to the frequency of fires of varying severity, based on long-term (19-year) vegetation and fire monitoring data from Kakadu, Litchfield and Nitmiluk National Parks. The model is described by equations for lowland and upland situations:

∆TC_lowland_ = 1.018 – 0.0143 * ln(TC_initial_) + 0.0005 * MAR – 0.0072 * FLS – 0.0382 * FMS – 0.1257 * FHS (Eqn 2)

∆TC_upland_ = 1.015 – 0.0143 * ln(TC_initial_) + 0.0005 * MAR – 0.0072 * FLS – 0.0382 * FMS – 0.1257 * FHS (Eqn 3)

where ∆TC_lowland_ and ∆TC_upland_ are the proportional change in the carbon stock in above-ground live tree biomass (% year^-1^) in lowland and upland areas, respectively, TC_initial_ is the starting carbon stock in above-ground live tree biomass (t C ha^-1^), MAR is mean annual rainfall (mm), FLS is the frequency of low severity fires (fires year^-1^), FMS is the frequency of moderate severity fires (fires year^-1^), FHS is the frequency of high severity fires (fires year^-1^), with the three severity classes following [[9](#_ENREF_9)].

The frequencies of the fire severity classes was calculated from the seasonal distribution of each fire severity class (from [[9](#_ENREF_9)]: Table 1a), and the seasonal proportion burnt (%EDS or %LDS):

FLS = %Low(EDS) * %EDS + %Low(LDS) * %LDS (Eqn 4)
FMS = %Mod(EDS) * %EDS + %Mod(LDS) * %LDS (Eqn 5)
FHS = %High(EDS) * %EDS + %High(LDS) * %LDS (Eqn 6)

where %Low(EDS) is the proportion of low severity fires occurring in the early dry season, and so forth.

We used the model of [[6](#_ENREF_6)] to simulate, using an annual time-step, carbon stock in above-ground live tree biomass, in response to varying fire management regimes (i.e. baseline vs. IFM). We ran the simulation over a 25-year period, assuming a starting carbon stock in above-ground live tree biomass of 25.1 t C ha^-1^ and mean annual rainfall of 1,313 mm. After 25 years, we calculated the difference in carbon stock in above-ground live tree biomass (t C ha^-1^) between the baseline and IFM scenarios. This was converted to t CO_2_-e ha^-1^ , accounting for the CO_2_ / C mass ratio (44/12), annualised and extrapolated over the given region.

**Results**

*Emissions abatement*

The difference between baseline (1995-2004) and IFM (2005-14) emissions for WALFA is given in Table 2a. The realised mean annual abatement for WALFA was 0.06 and 0.05 t CO_2_-e ha^-1^, for lowland and upland units respectively, yielding a total mean annual abatement of 140,000 t CO_2_-e. Baseline (2005-14) and, based on relative parity with IFM under WALFA, potential annual emissions abatement for the three national parks were 59,433 t CO_2_-e for Kakadu, 8,301 t CO_2_-e for Litchfield, and 18,702 t CO_2_-e for Nitmiluk (Table 2b).

*Carbon sequestration in fine and coarse fuel*

The baseline mean annual FCWF sequestration (t CO_2_-e ha^-1^ y^-1^) was calculated as 0.53 for WALFA, 0.47 for Kakadu, 0.43 for Litchfield, and 0.46 for Nitmiluk. Mean annual FCWF sequestration (t CO_2_-e ha^-1^ y^-1^) for WALFA under IMF was calculated as 0.69, and potentially under equivalent IMF conditions, 0.70 for Kakadu, 0.67 for Litchfield, and 0.68 for Nitmiluk. These quanta are 3.5, 4.4 and 6.0 times the potential emissions abatement achievable for lowland units in Nitmiluk, Litchfield and Kakadu, respectively. For lowlands and uplands collectively, the equivalent quanta are 3.2, 3.7 and 5.5 times the potential emissions abatement in respective parks.

*Carbon sequestration in above-ground tree biomass*

The mean fire severity for the 10 year baseline calculated for 25 years produced lowland tree carbon sequestration (t CO_2_–e ha^-1^ yr^-1^) of 0.12 for KNP, -0.21 for Litchfield, and 0.27 for Nitmiluk. For WALFA, baseline sequestration was 0.15 (t CO_2_–e ha^-1^ yr^-1^), but under IFM it was 0.61 (t CO_2_–e ha^-1^ yr^-1^) in the lowlands and 0.31 (t CO_2_–e ha^-1^ yr^-1^) in the uplands. The potential sequestration for the three parks was similar under IFM and overall produced credits 8.5, 4.8 and 4.5 times the emissions abatement for Kakadu, Litchfield and Nitmiluk, respectively.

**Table S2: (a) Emissions abated from WALFA for baseline (1995-2004) and under Improved Fire Management (IMF) period (2005-2014), (b) estimated potential abatement for the three national parks, assuming enhanced fire management as achieved for WALFA**

| **Site** | **Landscape unit** | **Area** | **Mean baseline emissions per site** | **Mean Baseline emissions per unit area** | **Mean IFM emissions** | **Mean IFM emissions per unit area** | **Mean Abatement per site** | **Mean Abatement per unit area** |
| --- | --- | --- | --- | --- | --- | --- | --- | --- |
|  |  | **(ha)** | **(t CO_2_-e y-^1^)** | **(t CO_2_-e ha^-1^ y-^1^)** | **(t CO_2_-e y-^1^)** | **(t CO_2_-e ha^-1^ y-^1^)** | **(t CO_2_-e y-^1^)** | **(t CO_2_-e ha^-1^ y-^1^)** |
| **(a) WALFA** | Lowland | 2,055,131 | 347,584 | 0.17 | 226,229 | 0.11 | 121,356 | 0.06 |
|  | Upland | 392,650 | 50,037 | 0.13 | 31,458 | 0.08 | 18,578 | 0.05 |
| **(b) Parks** | |  |  |  |  |  |  |  |
| **Kakadu** | Lowland | 1,506,144 | 224,110 | 0.15 |  |  | 58,314 | 0.04 |
|  | Upland | 98,538 | 9,014 | 0.09 |  |  | 1,119 | 0.01 |
| **Litchfield** | Lowland | 106,881 | 18,392 | 0.17 |  |  | 6,626 | 0.06 |
|  | Upland | 20,506 | 3,318 | 0.16 |  |  | 1,675 | 0.08 |
| **Nitmiluk** | Lowland | 246,231 | 43,825 | 0.18 |  |  | 16,720 | 0.07 |
|  | Upland | 23,856 | 3,894 | 0.16 |  |  | 1,982 | 0.08 |

**References**

1. Fisher R, Edwards AC. Fire extent mapping: procedures, validation and website application. In: Murphy BP, Edwards AC, Meyer CP, Russell-Smith J, editors. Carbon Accounting and Savanna Fire Management. Melbourne: CSIRO Publications; 2015. pp. 57-72.

2. ESRI. ArcGIS Desktop: Release 10. Redlands, CA: Environmental Systems Research Institute; 2011.

3. ESRI. ARC/INFO Version 9.2. Redlands, CA: Environmental Systems Research Institute; 2006.

4. CoA (Commonwealth of Australia). Carbon Farming (Reduction of Greenhouse Gas Emissions through Early Dry Season Savanna Burning) Methodology Determination. Canberra: Department of Climate Change & Energy Efficiency, Australian Govt; 2012. Available: <http://www.comlaw.gov.au/Details/F2012L01499>. Accessed 10 April 2015].

5. Cook GD, Liedloff AC, Murphy BP. Towards a methodology for increased carbon sequestration in dead fuels through implementation of less severe fire regimes in Savannas. In: Murphy BP, Edwards AC, Meyer CP, Russell-Smith J, editors. Carbon Accounting and Savanna Fire Management. Melbourne: CSIRO Publications; 2015. pp. 321-326.

6. Cook GD, Liedloff AC, Murphy BP. Predicting the effects of fire management on carbon stock dynamics using statistical and process-based modelling. In: Murphy BP, Edwards AC, Meyer CP, Russell-Smith J, editors. Carbon Accounting and Savanna Fire Management. Melbourne: CSIRO Publications; 2015. pp. 295-315

7. Murphy BP, Russell-Smith J, Prior LD. Frequent fires reduce tree growth in northern Australian savannas: implications for tree demography and carbon sequestration. Global Change Biology 2010; **16**: 331-343.

8. Edwards A, Russell-Smith J, Meyer CP. Contemporary fire regime risks to key ecological assets and processes in north Australian savannas. International Journal of Wildland Fire 2015; - DOI: <http://dx.doi.org/10.1071/WF14197>.

9. Russell-Smith J, Edwards AC. Seasonality and fire severity in savanna landscapes of monsoonal northern Australia. International Journal of Wildland Fire 2006; 15: 541-550.

10. CoA (Commonwealth of Australia). Carbon Credits (Carbon Farming Initiative—Emissions Abatement through Savanna Fire Management) Methodology Determination 2015. Canberra: ComLaw, Australian Govt; 2015. Available: <http://www.comlaw.gov.au/Details/F2015L00344>. Accessed 10 April 2015].

11. Thackway R. A vegetation fuel type map for Australia’s northern savannas. Canberra: Department of the Environment; 2014.
